# International recommendations for outpatient palliative care and prehospital palliative emergencies – a prospective questionnaire-based investigation

**DOI:** 10.1186/1472-684X-12-10

**Published:** 2013-02-21

**Authors:** Christoph HR Wiese, Christoph L Lassen, Utz E Bartels, Mahmoud Taghavi, Saleem Elhabash, Bernhard M Graf, Gerd G Hanekop

**Affiliations:** 1 Department of Anaesthesiology, University Medical Centre Regensburg, Franz-Josef-Strauß-Allee 11, Regensburg 93053, Germany; 2 Department of Anaesthesiology and Intensive Care Medicine, University Medical Centre Ulm, Ulm, Germany; 3 Department of Anaesthesiology, Emergency and Intensive Care Medicine, University Medical Centre Goettingen, Goettingen, Germany

**Keywords:** Specialised palliative care services (SPCS), Emergency medical care, Palliative care, Palliative emergency, End-of-life care

## Abstract

**Background:**

To determine the international recommendations and current practices for the treatment and prevention of palliative emergencies. The primary goal of the study was to gather information from experts on their nationally practised concepts.

**Methods:**

One hundred and fifty self-report surveys were distributed by email to selected leading experts (palliative and emergency medical care) in Europe, North and South America, Africa, Asia, and Australia. An expert in this context was defined as an author of an article that was ranked by three reviewers as relevant to outpatient palliative and emergency medical .

**Results:**

The total response rate was 61% (n = 92 experts). Survey responses were obtained from 35 different countries. The following standards in the treatment of palliative emergencies were recommended: (1) early integration of “Palliative Care Teams” (PCTs) and basic outpatient palliative care systems, (2) end-of-life discussions, (3) defined emergency medical documents, drug boxes, and “Do not attempt resuscitation” orders and (4) emergency medical training (physicians and paramedics).

**Conclusions:**

This study detected structurally and nationally differences in outpatient palliative care regarding the treatment of palliative emergencies. Accordingly, these differences should be discussed and adapted to the respective specifications of individual single countries. A single established outpatient palliative emergency medical care concept may be the basis for an overall out-of-hospital palliative care system.

## Background

“Specialised Palliative Care Services” (SPCS) are an important element in the treatment of outpatient palliative emergencies [1-3]. A legal basis for receiving outpatient palliative care is outlined in the recommendation 24 of the Committee of Ministers to the European member states on the organisation of palliative care (adopted by the Committee of Ministers on 12 November 2003 at the 86^th^ meeting of the Minister Deputies Council of Europe): “Specialized palliative care should be available for all patients when they need it, at any time and in any situation”.

SPCS should increase patients’ satisfaction and provide adequate palliative care which may allow the patient to safely remain at home, which may lead to safety at home [2,4,5]. This care would include prevention, if possible, and preparedness to respond to emergency situations at home either by the team or by other means [4,6-12]. Patients in a palliative care setting and their care-giving relatives define good symptom control and security [for example, by access to 24-hour outpatient medical palliative care teams (PCTs)] and nursing care as major issues in outpatient palliative care [2,5,6]. Therefore, adequate SPCS should offer a patient satisfaction and safety in a home-based care environment [1,2,5]. Furthermore, through SPCS, the provision of adequate symptom control reduces a patients’ anxiety in emergency situations [8,10].

However, both nationally and internationally, only minimal financing exists for out-of-hospital care. Therefore, outpatient PCTs are only partially guaranteed. For this type of care, other medical care systems are accessed for emergency medical treatment (3-10% of all prehospital emergencies are defined as palliative emergencies) of out-of-hospital palliative care emergencies (for example, prehospital emergency medical systems (EMS); paramedics and/or prehospital emergency physicians) [1,13-17]. This suggests that, theoretically, every prehospital emergency physician (EP), paramedic, and in-hospital emergency physician (ED) may be confronted by patients in a palliative care situation at an advanced stage of their disease who need medical care.

The aim of the present investigation was to determine international recommendations for the treatment and prevention of palliative emergencies (outpatient palliative care and/or emergency medical care systems) using a prospective questionnaire-based design. Additionally, the differences among national and regional recommendations were also investigated and discussed. Based on the results, the authors define current practices in regard to the future care of palliative emergencies.

## Methods

### Literature analysis and selection of the experts

A total of 100 international experts in palliative and emergency medical care were selected by an analysis of the literature. Using defined free-text search terms and key words (“palliative medicine” and “emergency medicine”, “palliative emergency”, “end of life decision”, “CPR” and “palliative care”, “palliative care” and “emergency medicine”), three independent reviewers conducted a search and the review of the PubMed, Medline, EMBASE medical. An expert in this context was defined as an author of an article that was ranked by all three of the reviewers as relevant to SPCS and emergency medical care.

The literature search yielded an overabundance of West-European, American and Canadian experts. Therefore, 50 “national associations for palliative care opinion leaders” were also invited to participate in the present investigation. However, preference was given to the selected experts and their published articles and work, rather than first asking the national societies to identify experts in this research field.

The survey employed consisted of both closed questions (for example, Likert-scale and multiple-choice formats) and the option for individuals to comment (see: subsection example of parts of the used questionnaire; mixed methods design, self-administered design). Data were collected using this researcher-developed data coding-form/questionnaire for five types of information: (1) demographic data, (2) in-hospital palliative care structures, (3) out-of-hospital palliative care structures, (4) concepts in outpatient palliative emergency medical care, and (5) advance directives/end-of-life decisions.

### Example of parts of the used questionnaire

The objectives of the demographics and descriptive research were to obtain information on the following:

• The expert’s primary profession.

• The number of inhabitants of the town and surrounding area the expert works

• The type of hospital where the expert works.

• The expert’s definition of palliative care.

• The established palliative care systems (for example, a palliative ward, in-hospital palliative care system, out-of-hospital palliative care system) in the area the expert works in.

• The medical department responsible for the palliative care system.

• The types of staff involved in the care of palliative care patients (nursing and physician staff).

• The prehospital emergency care system.

• The percentage of out-of-hospital palliative care emergencies.

• The integration of out-of-hospital palliative care teams into the prehospital emergency care of palliative care patients.

• The integration of palliative care medicine into the education of prehospital emergency staff.

• The types of cooperation between the palliative care teams/palliative care medicine and emergency medical staff/emergency medicine.

The specific research objectives were to determine the following:

• Nationally and regionally recommended concepts for the treatment of palliative emergencies.

• The integration of specialised outpatient palliative care teams into the primary emergency treatment concerning crises in palliative care patients.

• The existing cooperation between palliative care and emergency medical care physicians.

• The emergency medical staff’s education concerning palliative care and “end-of-life” decisions.

• The provision of advance directives and DNAR orders.

• The approach to the use of emergency drugs in a home-care setting (for example, drug boxes and the education of caregivers concerning the use of these drugs).

• The integration of palliative care concepts into emergency medical departments and intensive care units.

• The development of an outpatient concept concerning crises in palliative care patients based on the internationally mentioned systems.

The definition of a “patient in a palliative care situation” and “palliative care” was implemented using the international classification of the European Association for Palliative Care (EAPC 2010):

*“Palliative care is the active, total care of the patients whose disease is not responsive to curative treatment. Control of pain, of other symptoms, and of social, psychological and spiritual problems is paramount. Palliative care is interdisciplinary in its approach and encompasses the patient, the family and the community in its scope. In a sense, palliative care is to offer the most basic concept of care – that of providing for the needs of the patient wherever he or she is cared for, either at home or in the hospital. Palliative care affirms life and regards dying as a normal process; it neither hastens nor postpones death. It sets out to preserve the best possible quality of life until death.”* [EAPC 2012]

### Data extraction and analysis

One hundred and fifty self-report surveys were distributed by email to selected leading experts in Europe, North and South America, Africa, Asia, and Australia. The primary investigation was open to participants for six months.

The specific research objectives were to determine (1) internationally recommended concepts for the treatment of prehospital palliative emergencies and (2) the development of an outpatient concept for the treatment of critically situations in palliative care patients. The data were recorded with the MS Excel 2003 calculation program (Microsoft Inc®, Seattle WA, USA). Descriptive statistics were used for the respondent, palliative and emergency care characteristics. Participants’ data were stored in a database, and a statistical analysis was performed using SPSS version 16.0 (SPSS Inc®, Chicago Ill, USA) where necessary.

Consent from the participants obtained either by the return by mail of the questionnaire or was online based. The appropriate data protection guidelines and ethical principles were adhered to according to #26 of the Declaration of Helsinki. The institutional review board approved the study, and approval was also granted by the local ethical commission (University of Goettingen and University of Regensburg, Germany).

Pilot testing of the questionnaire with 10 participants for comprehension and usability was conducted prior to its administration. No changes were made as a result of the pilot testing.

## Results

The total response rate was 61% (n = 92 experts). Survey responses were obtained from 35 different countries. Groups were divided into five continents. Europe was divided into Western and Eastern Europe. America and Australia were divided into North and South (Tables 1 and 2).

**Table 1:** Demographics

**Profession (more than one possible)**	**Total number (%)**	**Hospital level**	**Total number (%)**
**Internal medicine**	51 (55)	**Level 1**	52 (57)
**Anaesthesiology**	13 (14)	**Level 2**	27 (29)
**Surgery**	6 (7)	**Level 3**	8 (9)
**Palliative medicine**	52 (57)	**Outpatient care**	5 (5)
**Prehospital Emergency Medicine**	2 (2)		
**Number of inhabitants in the town you are working in**		**Number of inhabitants in the surrounding area**	
**<50.000**	8 (9)	**<50.000**	0 (0)
**50.001-100.000**	8 (9)	**50.001-100.000**	4 (4)
**00.001-250.000**	13 (14)	**100.001-250.000**	19 (21)
**250.001-500.000**	26 (28)	**250.001-500.000**	10 (11)
**00.001-1.000.000**	19 (21)	**500.001-1.000.000**	32 (35)
**>1.000.000**	18 (19)	**>1.000.000**	27 (29)
**Hospitals in the surrounding area of 50 km**			
**1-5**	29 (32)		
**6-10**	33 (36)		
**11-15**	26 (28)		
**>15**	4 (4)		
	**Answered questionnaires [total number (%)]**	**Total number of all mailed questionnaires**	
**North America**	25 (50)	50	
**South America**	3 (60)	5	
**Western Europe**	28 (62)	45	
**Eastern Europe**	22 (88)	25	
**Africa**	4 (80)	5	
**Asia**	5 (50)	10	
**Australia (North and South)**	5 (50)	10	
**Total**	**92 (61)**	**150**	

**Table 2:** Included countries

**North America**	**Europe**	**Africa**	**Asia**	**Australia**
Canada	**Western Europe**	Kenya	China	North Australia
United States	Belgium	South Africa	India	South Australia
**South America**	Denmark	Uganda	Japan	
Argentina	France			
Brazil	Finland			
Chile	Germany			
	Greece			
	Italy			
	Netherlands			
	Norway			
	Portugal			
	Spain			
	Sweden			
	United Kingdom			
	**Eastern Europe**			
	Bulgaria			
	Czech Republic			
	Kazakhstan			
	Latvia			
	Lithuania			
	Poland			
	Romania			
	Russia			
	Serbia			

Twenty-three percent (n = 21) of the experts defined cancer patients as patients in a palliative care situation. Seventy-seven percent (n = 71) defined patients in a noncurative stage of their disease independent of its type as patients in a palliative care situation.

The palliative medical care structures that were described were divided into only in-hospital structures (23%), only outpatient “Palliative Care Teams” (PCT) (2%), in-hospital and outpatient palliative care structures (65%), and no available palliative care structures (10%).

Fifty-five percent (n = 51) of the investigated participants worked actively in palliative care systems and/or departments. Two were experts in prehospital emergency medicine. A prehospital emergency-physician-based EMS was reported by 33 experts (36%). A paramedic-based EMS was reported by 54 experts (59%). Five experts (5%) reported no existing EMS. The respondents reported the percentage of all prehospital emergencies that were palliative emergencies to be approximately (1) <5% (13%, n = 12), (2) 5% (29%, n = 27), (3) 10% (44%, n = 40), and (4) more than 10% (14%, n = 13).

In the open-ended answers, the following standards in palliative emergency medical care were considered to be important:

(1) SPCS structures and basic palliative care treatment: 91% of the respondents (n = 84) explicitly stated the necessity for 24-hour accessibility to SPCS (e.g., the simultaneous alert of the EMS and PCT, the alert of the PCT by the EMS, and the primary alert of the PCT by the emergency operations centre).

(2) Structural cooperation between palliative and prehospital emergency physicians: 70% of the respondents (n = 64) rated the early cooperation between palliative and prehospital emergency medical staff (prehospital emergency physicians as well as paramedics) as necessary. However, this cooperation is currently very limited (2%). Because not all countries have SPCS, such cooperation is still necessary to ensure the treatment of palliative emergencies.

(3) Medical education of emergency-medical staff concerning palliative care and “End-of-life” care: 92% of the questioned experts (n = 85) recommended the addition implementation of palliative care to emergency medical training curricula. However, an already existing structured training for emergency medical staff in “end of life” care was mentioned twice.

(4) “End-of-life” discussions between the patients’ trusted family caregivers and physicians: 89% of the respondents (n = 82) stated that “End-of-life” conversations and emergency preparation in an early stage of a life-limiting disease were necessary.

(5) Establishment of advance directives and “Do not attempt resuscitation” (DNAR) orders: The establishment of advance directives should potentially be integrated into the specific legislations of different countries according to 60% of the questioned experts (n = 55). However, the experts were unable to provide consistent recommendations because of the existing national differences in legislation. Of particular importance, a total of 43 experts (47%) considered the obligatory introduction of DNAR orders for the special case of cardio-pulmonary resuscitation to be necessary.

(6) Preparing and involving trusted family caregivers in responding to emergency situations (including drug boxes and structured emergency medical care documents): 92% of the experts questioned (n = 85) considered the involvement of family caregivers to be essential in the treatment of palliative emergencies. There were different descriptions concerning the involvement of family members in different countries (for example, it is worthwhile to note the meaning of patient care to family members in African countries).

(7) The integration of palliative medical competence into the emergency department and intensive care units: Approximately 60% of the experts questioned (n = 56) recommended the integration of palliative medical competence into the emergency department and into intensive care units. Such integration should reduce burdensome and questionable therapeutic medical measures for palliative care patients and restrict the symptom control focused treatment of such patients. The presence of such expertise was reported by only emergency department experts and eight intensive care unit experts.

## Discussion

The participants in the investigation of SPCS reported it to be an important structure in the care of palliative emergencies in a home-care setting [1-4]. In many countries, this structure is still in need of development and expansion (for example, where there is a lack of financing or not enough qualified personnel for its implementation) [1].

In our research, we were able to show that the experts questioned were aware of the above-mentioned problem and that a demand for SPCS exists. Furthermore, the strengthening of cooperation between SPCS, general practitioners, and prehospital emergency medical staff is recommended [4,18,19]. This was previously recommended in an Australian review paper in 2003 [19]. This collaboration is important because of the limited exposure to their family physicians of patients who are in a palliative care situation and or in advanced stage of their disease [19]. The specialised support offered by PCTs is regarded to be necessary [19]. Further work provides evidence that patients in a palliative care situation who receive PCT rarely use emergency medical structures [1]. According to the results of our research, the development and improvement of the SPCS structure and the expansion of outpatient palliative medical basic care is the most effective way to reduce emergency medical treatment for outpatient patients in a palliative care situation. Unfortunately, the results of the present investigation describe problems in the provision of good palliative care and the treatment of palliative emergencies that are already relatively well known. Nevertheless, this type of care should be practiced, although it is currently practiced in all of the included countries.

The reply rate of the experts who were questioned was very high and detailed, particularly because of the questionnaire’s open response options. The care of patients in a palliative care situation at the end of life has different structural requirements for multiple professions [18,20,21]. Our research presents international recommendations for the structural cooperation between palliative and emergency medical care [18,22-24].

The following section discusses a way to optimise the care of patients in a palliative care situation in an outpatient emergency setting using the available systems and represents the recommendations of the experts that we questioned.

(1) The extension of SPCS and basic palliative care structures [4,25,26].

(2) The additional integration of outpatient SPCS into the emergency care provided by the emergency medical service, thus ensuring available outpatient care [25,26].

(3) Improvement in the communication and cooperation between PCT and emergency medical services [25,26].

(4) Enhancement of the medical education of emergency medical staff [14,15,22,23].

(5) Early implementation of “End-of-Life” conversations to define a palliative care patient [27-29].

(6) Generating the patient’s advance directive for specific palliative emergencies [20,21,28].

(7) Preparing trusted family caregivers, outpatient nursing service and nursing homes for palliative emergencies and constructing emergency plans for their use [4,9,18,30].

(8) Distribution of emergency drug boxes that can be used independently by all persons who are integrated into the care of the patient [31].

(9) Integration of palliative care and ethical competence into Intensive Care Units und Emergency Departments [3,32-34].

It is also important to note here the existence of relevant worldwide differences in emergency systems that can be significant in the care of patients in a palliative care setting who are in acute emergency situations [35]. These differences were noted by the experts questioned and would be partially integrated into outpatient palliative care. Experts, particularly in countries with paramedic-based emergency systems, recommended physician-supported SPCS. The possible disadvantage of a paramedic-based emergency system was described in the literature, particularly in regard to DNAR orders. In this context, clear differences, also within the different paramedic-based emergency systems, were reported. The objective of this research was not to assess the different emergency medical care systems. However, in the future it may be helpful to discuss possible general problems in the care of outpatient palliative care patients in acute emergency situations on the basis of functioning emergency medical systems and suggest possible solutions. Furthermore, it may be necessary to define general problems that may arise in the care of outpatient palliative care patients in emergency situations, or palliative emergencies, on the basis of the reported international systems and to suggest possible solutions.

### Limitations of the study

Our investigation has a number of limitations. The interview design was a substantial limitation. The closed-question section of the interview did not include all of the same aspects as the open-question section. Furthermore, there is no clear consensus on how to report and analyse Likert responses. Our report only considers the recommendations that were voluntarily reported. However, options from many of the experts who responded to the interview questions were of use for our study. In the search for experts with published articles that were considered relevant to palliative care and emergency medical care, the goal was to obtain their opinions on nationally and regionally recommended concepts. We must also mention that it is questionable whether the selected experts’ opinions are representative because the questions were addressed only by experts in their fields. This may be a potential bias of our investigation. It is likely that experts who have published papers are aware of aspects of excellence in their local service provision. Representatives of national organisations may not be aware of these local developments, but they will be aware of national perspectives and drivers of change, bringing different strengths and perspectives to the questions. However, these limitations did not alter the objectives of our study. Many of the answers and recommendations that were volunteered by the experts were significantly evaluated and could be considered independently of their frequent reporting in the development of an optimal outpatient palliative medical care model for emergency situations. Another limitation is the focus on experts from Europe and the USA. Countries that have varied health care policies and palliative care structures can only benefit to a limited extent from the discussions of the development and optimisation of outpatient palliative medical emergency structures. There may be a “continent effect” in our investigation. However, the direct comparisons of the results between different countries are challenging because the methods used were not sufficiently similar, and most medical systems were restricted to particular specialties or specific palliative care systems. The purpose of our study was not to establish a worldwide standard to treat palliative emergencies but to determine different possibilities for functioning outpatient palliative medical emergency structures and to discuss their approaches in general. Finally, the implementation of these ideas is the responsibility of the individual countries, independent of their existing health care policies, structures and potentials.

## Conclusions

SPCS is well known worldwide and is a desired approach in the outpatient care of palliative patients in an end of life stage. An overview of many of the experts questioned describes a theoretically optimal structure for the care of palliative emergencies. However, it is essential to discuss these recommendations in national palliative organisations. Consequently, recommended care models should undergo further clinical studies to be validated.

## Competing interests

The authors declare that there are no conflicts. Parts of this investigation were given as an oral presentation at the Research Congress of the European Association for Palliative Care in Glasgow, Scotland 2010.

## Authors’ contributions

CHRW, UEB, MT, and GGH participated in designing the study. CHRW and UEB participated in collecting and entering the data. SE, BMG, CLL and GGH supported editing the manuscript. SE, BMG co-wrote the manuscript and added important comments to the paper. All authors read and approved the final manuscript.

## Pre-publication history

The pre-publication history for this paper can be accessed here:

http://www.biomedcentral.com/1472-684X/12/10/prepub
